# Two Chitotriose-Specific Lectins Show Anti-Angiogenesis, Induces Caspase-9-Mediated Apoptosis and Early Arrest of Pancreatic Tumor Cell Cycle

**DOI:** 10.1371/journal.pone.0146110

**Published:** 2016-01-21

**Authors:** Ruby Singh, Laxman Nawale, Dhiman Sarkar, C. G. Suresh

**Affiliations:** 1 Biochemical Sciences Division, CSIR-National Chemical Laboratory, Pune, India; 2 Combichem-Bioresource Center, CSIR-National Chemical Laboratory, Pune, India; IDI, Istituto Dermopatico dell'Immacolata, ITALY

## Abstract

The antiproliferative activity of two chito- specific agglutinins purified from *Benincasa hispida* (*Bh*L) and *Datura innoxia* (*Di*L9) of different plant family origin was investigated on various cancer cell lines. Both lectins showed chitotriose specificity, by inhibiting lectin hemagglutinating activity. On further studies, it was revealed that these agglutinins caused remarkable concentration-dependent antiproliferative effect on human pancreatic cancerous cells but not on the normal human umbilical vein endothelial cells even at higher doses determined using MTT assay. The GI_50_ values were approximately 8.4 μg ml^-1^ (0.247 μM) and 142 μg ml^-1^(14.8 μM) for *Bh*L and *Di*L9, respectively, against PANC-1 cells. The growth inhibitory effect of these lectins on pancreatic cancer cells were shown to be a consequence of lectin cell surface binding and triggering G_0_/G_1_ arrest, mitochondrial membrane depolarization, sustained increase of the intracellular calcium release and the apoptotic signal is amplified by activation of caspases executing cell death. Interestingly, these lectins also showed anti-angiogenic activity by disrupting the endothelial tubulogenesis. Therefore, we report for the first time two chito-specific lectins specifically binding to tumor glycans; they can be considered to be a class of molecules with antitumor activity against pancreatic cancer cells mediated through caspase dependent mitochondrial apoptotic pathway.

## Introduction

For decades, DNA used to be the main target of anticancer drugs, but due to reoccurrence of resistance the molecular targeted therapy with molecules having specificity and selectivity such as lectins have shown better promise for the cancer treatment. These lectins are proteins that can bind saccharide containing moieties in a reversible manner using direct interaction or through water bridges and facilitated by oligomerization and post translational modifications [1]. Recently, plant lectins have attracted much attention due to their ability to trigger various biological processes, such as cell agglutination, immunomodulation along with possessing antimicrobial and anti-viral activities. In addition to these properties, some lectins can recognize tumor associated glycans and therefore can differentiate malignant cells from normal cells based on the degree of glycosylation associated with metastasis. Due to lectin’s specificity and selectivity property, they are known to induce cytotoxicity to tumor cells [2]. Extensive research done to characterize new plant lectins has expanded lectin classification from 7 families to 12 families [3]. Among the major lectin families, legume and type 2 ribosome inactivating proteins (RIPs) lectins have received more attention due to their remarkable antineoplastic activity. For instance, a legume lectin like ConA possesses anti-tumor activity by inducing apoptosis and autophagy [4] and mistletoe lectins (MLs), a type 2 RIPs induce apoptosis via mitochondria/ death receptor pathways [5, 6]. MLs act as adjuvant agent reducing the treatment associated side effects in chemotherapy and radiotherapy in pre-clinical trials [7].

Any deregulation in the apoptosis process causes cancer, autoimmune diseases, bone marrow rejection, neuro-degenerative disorders and many such disorders. Apoptosis, a type I programmed cell death (PCD) is an evolutionary conserved process which is characterized by cytoplasmic and nucleic condensation, DNA fragmentation, membrane blebbing and phagocytosis [8]. The two major pathways of apoptosis are distinguished by the involvement or not of caspases, while mitochondria connect the different pathways as cross-talk intermediate. The caspase cascade is triggered by the depolarization of mitochondrial membrane releasing cytochrome c and calcium ions which act as crucial secondary messenger, initiates the formation of apoptosomes leading to the cell death. Therefore, the molecules blocking the tumorigenesis cascade, like plant lectins can become an attractive strategy for modulating the components of cell death machinery via apoptotic pathway [9]. Many lectins have been purified and characterized for its anti-neoplastic activity like *Sclerotium rolfsii* [10], leczyme [11], *Rhizoctonia bataticola* [12] and wheat germ agglutinin (WGA) [13]. Tumor angiogenesis also plays critical role in tumor proliferation and spreading through metastasis [14]. Based on different anti-angiogenesis approaches, new anticancer strategy has also emerged and is undergoing extensive study in Phase I–III trials [15].

Among all cancers, pancreatic cancer is the most deadly type. It is ranking fourth among the leading causes of cancer death, behind lung, colorectal, and breast cancer. The five-year survival rate is 5% for all combined patients due to difficulties in its early detection. Surgery, radiotherapy and chemotherapy treatments are available for pancreatic cancer. However, there is no improvement in the survival rate and side effects are by no means inconsequential. Research for developing safer and effective therapies is required. The present study was undertaken to investigate the anticancer properties of two chito-specific lectins, *Benincasa hispida* lectin purified from ashgourd fruit (*Bh*L) and *Datura innoxia* lectin purified from datura seeds (*Di*L9). These agglutinins are structurally unrelated but bind to same sugar (chitotriose) with different affinities. Our recent studies have shown that they have strong killing effect on pancreatic cancerous cells (PANC-1, CFPAC-1 and MIA PaCa-2) *in vitro* at lower doses. Both the lectins induced apoptosis in these cells via caspase-dependent mitochondrial pathway and also inhibited angiogenic activity of endothelial cells.

## Materials and Methods

### Purification of lectins

*Bh*L was purified from fruit extract of *Benincasa hispida* using chitin affinity chromatography and eluted using 0.05 M Glacial acetic acid. *Di*L9 was purified from *Datura innoxia* seeds using Q-sepharose ion exchange column, followed by Sephacryl S-200 gel filtration chromatography for achieving final homogenous lectin preparation [16]. The lectin purity was confirmed using 12% SDS-PAGE and activity by hemagglutination assay using 3% rabbit’s erythrocyte suspension. All cell line studies were conducted using purified lectin preparations only. The lectin solutions were filter sterilized for cell line studies.

### Cell lines and culture conditions

The effect of lectins on cell growth was determined in a primary human umbilical vein endothelial cells (HUVECs), a mouse fibroblast cell line (L929; Passage No. 40), and in a panel of human tumor cells including lung adenocarcinoma (A549; Passage No. 37), acute monocytic leukemia cell line (THP-1; Passage No. 16) and pancreatic adenocarcinoma (PANC-1; Passage No. 29), Human pancreatic ductal adenocarcinoma cell line (CFPAC-1; Passage No.25), Human pancreatic epithelial carcinoma cell line (MIA PaCa-2; Passage No.19) and cervix adenocarcinoma (HeLa) obtained from the European Collection of Cell Cultures (ECCC, Salisbury, UK). HUVECs were maintained in M200 Media supplemented with 50X LVES (Gibco, Invitrogen); THP-1 was maintained in RPMI 1640; L929, A-549, PANC-1, CFPAC-1 and MIA PaCa-2 cells were maintained in Dulbecco’s Modified Eagle Medium (DMEM). HeLa and macrophages were cultured in Eagle's Minimum Essential Medium (EMEM). All media used were supplemented with 10% fetal bovine serum (FBS; Gibco) and the cells were maintained at 37°C and 5% CO_2_ in a humidified atmosphere.

### Cell growth inhibition assay

The *in vitro* cyto-toxic effects of lectins were determined by using reduction of 3-(4, 5-dimethylthiazol-2-yl)-2, 5- diphenyltetrazolium bromide (MTT) assay to produce formazan crystals [17]. An aliquot of 100 μl of each sub-confluent cell lines (cell density: 1x10^5^ cells ml^-1^) were seeded in 96-well flat bottom microtitre plate. The plates were incubated at 37°C in an atmosphere of 5% CO_2_ and 95% relative humidity within a CO_2_ incubator. After 24 h of incubation, the cells were treated with serial dilutions of lectins (*Bh*L and *Di*L9, 0.05–5.0 mg ml^-1^) and Carboplatin (1 mg ml^-1^, Sigma Chemicals, USA, positive control). At the end of the incubation, the cells were washed thrice with phosphate buffered saline (PBS) followed by the addition of 10 μl of MTT solution (5 mg ml^-1^, Sigma Chemicals, USA) in each well. After 4 h of incubation at 37°C, the formazan product was solubilized by the addition of 200 μl of acidified isopropanol and absorbance was measured on a SPECTRAmax PLUS 384 plate reader (Molecular Devices Inc, USA), at 570 nm. Percentage of cell viability was calculated with respect to untreated cells considered as 100% grown. All experiments were performed in triplicates, and the quantitative measurement was expressed as the average ± standard deviation.

### Microscopic observation of cell morphology

#### Acridine orange (AO) staining

Staining of cells with Acridine orange (AO) was performed to study cell death pattern induced by lectins after 24 h of incubation. Pancreatic cancer cells were selected for further studies based on the MTT assay (Table 1), where both the lectins (*Bh*L and *Di*L9) showed high inhibitory effect on cell growth at lower doses. Exponentially growing PANC-1 cells were seeded in a 96 well flat bottom plate and treated with lectins, *Bh*L and *Di*L9 at their GI_50_ values (50% growth inhibitory concentration), 0.247 and 14.8 μM, respectively for 24 h. The untreated cells were taken as control. The cells were collected by centrifugation and washed with PBS. Apoptotic nuclear morphology was visualized after fixing the cells with 3.7% paraformaldehyde and stained with AO (8.5 μg ml^-1^, Sigma Chemicals, USA) for 20 min in the dark. The cells were visualized under EVOS FL Cell Imaging System (Life technologies) using filter sets, 470 nm excitation, and 525 nm emission.

**Table 1:** Inhibitory effect of *Bh*L and *Di*L9 on different cell lines.

Cell name	*Bh*L		*Di*L9		^b^Carboplatin	
	^a^GI_50_	GI_90_	GI_50_	GI_90_	GI_50_	GI_90_
^*1^HUVEC	>130	>130	>520	>520	>10	>10
^#2^L929	>130	>130	>520	>520	>10	>10
^##3^THP-1	>208	>208	>1000	>1000	0.1374±0.53	5.8140±0.02
^##4^A549	29.32±0.23	200.54±2.08	344.04±0.56	>520	0.0035±0.71	0.0706±0.60
^##5^HeLa	34.09±0.15	168.45±0.55	146.01±0.35	>520	0.0048±0.36	0.075±0.56
^**##6**^**PANC-1**	**8.39±0.49**	**84.98±0.34**	**141.93±0.65**	**468.16±0.32**	**0.8519±0.96**	**5.7150±0.19**
^##7^CFPAC-1	11.32±0.45	172.81±0.25	86.49±0.15	913.32±0.35	0.968±0.46	8.76±0.36
^##8^MIA PaCa-2	13.99±0.4	186.13±0.5	67.92±0.45	943.13±0.5	0.376±0.36	5.0±0.66
^**9^Macrophage	58.52±1.32	>208	>1000	>1000	>10	>10

^a^ Growth Inhibition (GI): GI _50_ /GI _90_ (concentration which resulted in 50% /90% decrease in cell viability). Expressed in μg ml^-1^.

^b^ Standard anticancer drug and positive control.

*Primary cells: ^**1**^HUVECs- Human Umbilical Vein Endothelial Cells

^**#**^Cell Line from mouse origin: ^**2**^L929- areolar and adipose tissue fibroblast Cells

^**##**^Human cancer cell lines: ^**3**^THP-1 from acute monocytic leukemia, ^**4**^A549 from lung adenocarcinoma, ^**5**^HeLa from cervix adenocarcinoma, ^**6**^PANC-1 from pancreas carcinoma, ^7^ CFPAC-1 from pancreatic ductal adenocarcinoma, ^8^ MIA PaCa-2 from pancreatic epithelial carcinoma

**^9^Macrophage-PMA (phorbol myristate acetate)-differentiated human THP-1 macrophages.

#### Annexin V-FITC Apoptosis Assay

Pancreatic cancer cells were treated with lectins at their GI_50_ values (as mentioned before) for 2, 24, 48 and 72 h. After incubation, the cells were harvested and resuspended sequentially in binding buffer (0.01 HEPES, pH 7.4, containing 140 mM NaCl and 25 mM CaCl_2_) containing Annexin V- Fluorescein isothiocyanate **(**FITC, 3 μg ml^-1^, Sigma Chemicals, USA), 4′,6-Diamidino-2-phenylindole (DAPI, 1μM ml^-1^, Sigma Chemicals, USA) and Propium iodide (PI, 10 μg ml^-1^, Sigma Chemicals, USA) [18]. To determine the proportion of apoptotic and necrotic cells, the number of cells positive for Annexin V-FITC and PI were analyzed using a laser-scanning confocal microscope (LSCM), magnification 20X (Olympus FV1000) and 3D multichannel-image processing was done using Thermo Scientific High Content Screening (HCS) Studio 2.0 Cell Analysis Software.

### Cell Cycle Analysis

PANC-1 cells were treated with lectins at their GI_50_ values for 6, 12, 18 and 24 h, respectively. Cell cycle and sub G_0_/G_1_ distribution of cells were determined by staining with DAPI binding to DNA. The DNA content was measured using LSCM at 386 nm and the data was analyzed using HCS as described before for determining the cells in different phases of cell cycle [19].

### Detection of caspase activity

Activation of caspase-8, -9 and -3 in lectin stimulated pancreatic cancer cells was measured by caspase fluorimetric assay kit, according to the instructions given by the kit manufacturers. Apo Alert Caspase Luminescent Assay Kit (Promega, USA) and EnzChek Caspase-3 Assay Kit (Molecular probes, USA) were used for measuring caspase -8/-9 and -3 activity, respectively. Briefly, the cancer cells were treated with lectins and incubated for indicated time periods. The cells were lysed with lysis buffer at 4°C for 10 min, centrifuged and supernatant was collected. Supernatant samples (50 μl) were mixed with equal volumes of 2X reaction buffer and specific substrate conjugate (Z-DEVD–AMC) for caspase-3, acetyl-Ile-Glu-Thr-Asp p-nitroaniline (Ac-IETD-pNA) for caspase-8 and acetyl-Leu-Glu-His-Asp-p-nitroaniline (Ac-LEHD-p-NA) for caspase-9 provided by the kit manufacturers. 50 μl of the cell lysis buffer was taken as no-enzyme control to determine the background fluorescence of the substrate. After additions of substrates, the plates were incubated at 37°C for 1 h during which, the caspases cleave the substrates to release p-nitroaniline (p-NA). The role of caspase activation during apoptosis process was also studied by using caspase inhibitors. The fold increase in the activity of caspases was calculated by measuring the fluorescence intensities of the resulting product using a plate reader (VarioskanFlash, using SkanIt Software 2.4.5 RE, Thermo Scientific.) with 496/520-nm filters for caspase-3, 400/500-nm filters for caspase-8 and 380/460-nm filters for caspase-9.

### Measurement of cytoplasmic calcium release concentration [Ca^2+]^_i_

A fluorescent Ca^2+^ binding indicator, Fluo-4 acetoxymethyl ester (Fluo-4/AM, 4 μmol l^-1^, Invitrogen) was used to measure cytoplasmic [Ca^2+^] change by LSCM. In brief, the PANC-1 cells were treated with lectins at their GI_50_ values and incubated for 4, 8 and 12 h, respectively. After incubation, the plate was centrifuged at 200x g for 5 min and incubated with Fluo-4/AM for 30 min at 37°C. The cells were washed with cold PBS and fixed with 3.7% paraformaldehyde at 37°C for 15 min. The fixed cells were stained with DAPI (1 μM ml^-1^) and changes in level of calcium released were quantified by measuring the fluorescence intensity at a detection spectrum of 488 nm.

### Determination of Mitochondrial membrane potential (Δψ_m_)

After incubating the PANC-1, CFPAC-1 and MIA PaCa-2 cells treated with lectins for 4, 8 and 12 h, the cells were centrifuged and resuspended in 100 μl of DMEM loaded with Mito Tracker Red (0.1 μmol l^-1^, Invitrogen) incubated for 15 min at 37°C. Later, the cells were washed twice with PBS, fixed with 3.7% paraformaldehyde and the nuclei were stained with DAPI (1 μM ml^-1^). The images were acquired using a LSCM and analysed through HCS as already described. Alterations in the Δψ_m_ of lectin treated cells was quantified by the uptake of fluorochrome MitoTracker Red dye, a cationic cell permeant flurochrome that passively diffuses through plasma membrane of viable cells and readily get sequestered by mitochondria with active Δψ_m_ and have no cytotoxic effects [20]. A decrease in red fluorescence intensity was considered as an indication of mitochondrial membrane dysfunction.

### Anti-angiogenesis activity of lectins

To evaluate the anti-angiogenic activity of lectins, the three-dimensional tubular vessel formation by HUVECs, a widely used human endothelial cell line was used for the *in vitro* assay. 96-well culture plates were coated with Matrigel which was then allowed to solidify at 37°C for 1 h. HUVECs were washed, suspended in appropriate media, and added to Matrigel-coated wells (2.5 x 10^4^ cells per well), treated with the known pro-angiogenic compound, Vascular Endothelial Growth Factor (VEGF, Angiogenesis Starter Kit, Life technologies) and incubated to promote angiogenic tube formation. Cells were subsequently treated with lectins (*Bh*L: 0.247 and *Di*L9: 14.8 μM) and incubated for 12 h at 37°C in a 5% CO_2_ humidified atmosphere. Suramin (5 μg ml^-1^/4.0 μM, Sigma Chemicals, USA), a known anti-angiogenic agent was used as positive control. After 24 h of lectin treatment, the tubes were stained with rhodamine conjugated phalloidin which labels F-actin enabling the entire tube to be identified and the nuclei was stained with DAPI, enabling an estimate of the number of nuclei per tube. Automated imaging was done on Thermo Scientific Cellomics ArrayScan HCS Reader.

## Results

### Cytotoxic effect of lectins on primary and human cancer cells

In this study, we investigated the anticancer activity of two lectins, *Bh*L (34 kDa, homodimer) and *Di*L9 (9 kDa, monomer), purified from Cucurbitaceae and Solanaceae family, respectively. The lectin purification profile is shown in the **S1 Fig**. Both these lectins have chitotriose (N-acetylglucosmine oligomer) specificity, thermostable (upto 80°C) and stable at extreme pH range but widely vary in their structure as evidenced from biophysical studies. However, these lectins exhibit different affinity (K_a_, association constant) for the same chito sugar, with *Bh*L being more effective than *Di*L9.

**Fig 1:**
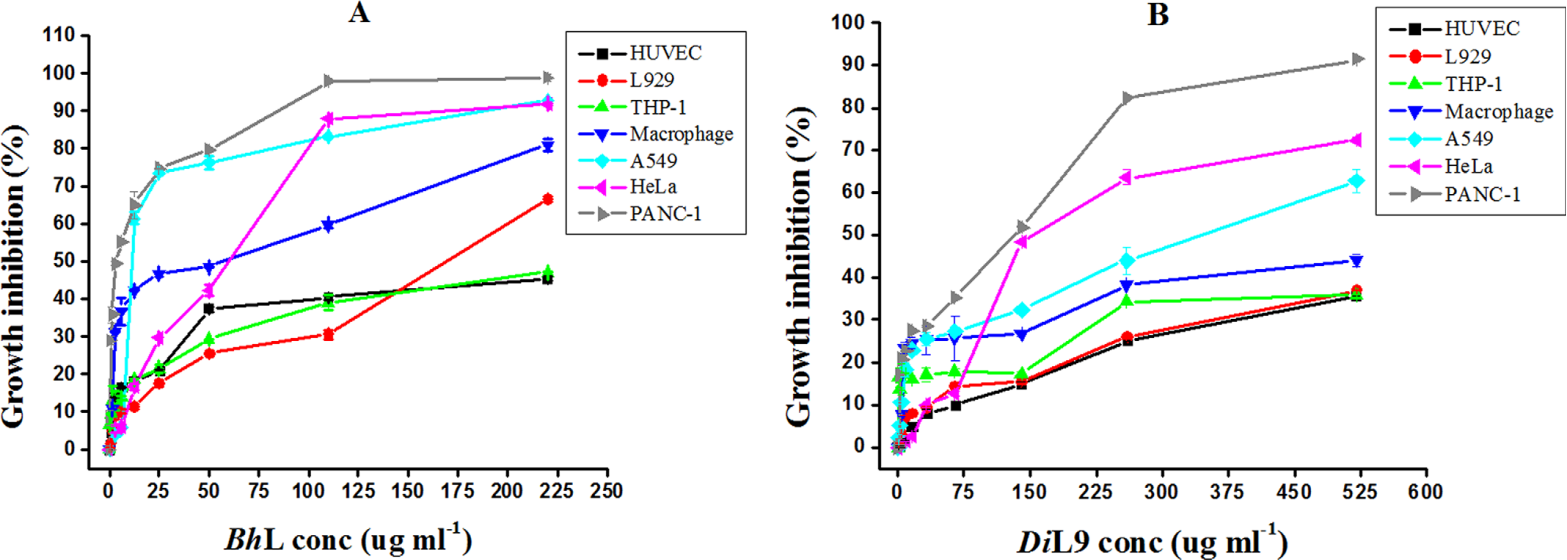
Effect of *Bh*L and *Di*L9 on proliferation and cell viability. Primary cells and different cancer cell lines were treated with serial concentrations of *Bh*L and *Di*L9 and incubated for 48 h. The growth inhibition (%) was measured by MTT assay by considering untreated cells as 100%. (A) Effect of *Bh*L and (B) *Di*L9 treatment on various cell lines. The values presented in the graph are the mean ± SD of two independent experiments done in triplicates.

The antiproliferative effect of *Bh*L and *Di*L9 on human primary cells and four cancer cell lines were evaluated by MTT assay. **Fig 1A and 1B**represents the percentage cytotoxicity shown by various cell lines on lectin treatment in a concentration-dependent manner after 48 h of exposure. However, the effect on cell viability was more pronounced in cultures treated with *Bh*L as per the MTT assay. Lectins showed no significant cytotoxicity towards HUVECs and L929 cells (<30% growth inhibition) even at higher concentration, demonstrating selectivity for tumor cells. After 48 h, GI_50_ of *Bh*L on the viability of PMA (phorbol myristate acetate)-differentiated human THP-1 macrophages was 58.52 μg ml^-1^ (1.7 μM) whereas *Di*L9 had nil effect. This might be due to differences in affinity of these lectins towards the cell surface glycans. 50% of cell growth cessation was achieved in the case of A549, HeLa and PANC-1 cells, but the lowest values of GI_50_ (*Bh*L: 8.4 μg ml^-1^/0.247 μM; *Di*L9: 142 μg ml^-1^/14.8 μM) and GI_90_ (*Bh*L: 85 μg ml^-1^/2.5 mM; *Di*L9: 468 μg ml^-1^/48.75 μM) were observed in the case of PANC-1 cells by both the lectins as shown in **Table 1**. The discrepancy in lectin cytotoxicity may be caused by the divergence of glycoprotein expression on different cell lines. Carboplatin (GI_50_: 0.85 μg ml^-1^/2.28 μM) exhibited potent cytotoxicity against all cell lines, as expected. To confirm lectin’s antiproliferative activity against human pancreatic cancer cell line, two different cancer cells were also tested, namely CFPAC-1 and MIA PaCa-2 of the same origin (**S2 Fig**). On comparing the GI_50_ and GI_90_ values among these three pancreatic cancer cells (Table 1), we found that both the lectins had comparatively higher inhibitory effect on PANC-1 cells.

The effect of these lectins on cancer cells was also estimated in the presence of 100% serum, to simulate the *in vivo* environments. For this, the lectins were pre-incubated with serum for 24 h and anti-proliferative activity was checked with MTT assay as described previously. 20% of growth inhibition was observed at higher concentration 1mg ml^-1^ (30 μM) of *Bh*L, whereas the effect of *Di*L9 was negligible (**S3 Fig**). This could be due to the binding of glycoproteins to the glycan binding regions of the lectins, thus inhibiting its activity [16].

### Lectin induced apoptosis in PANC-1 cells

**Fig 2:**
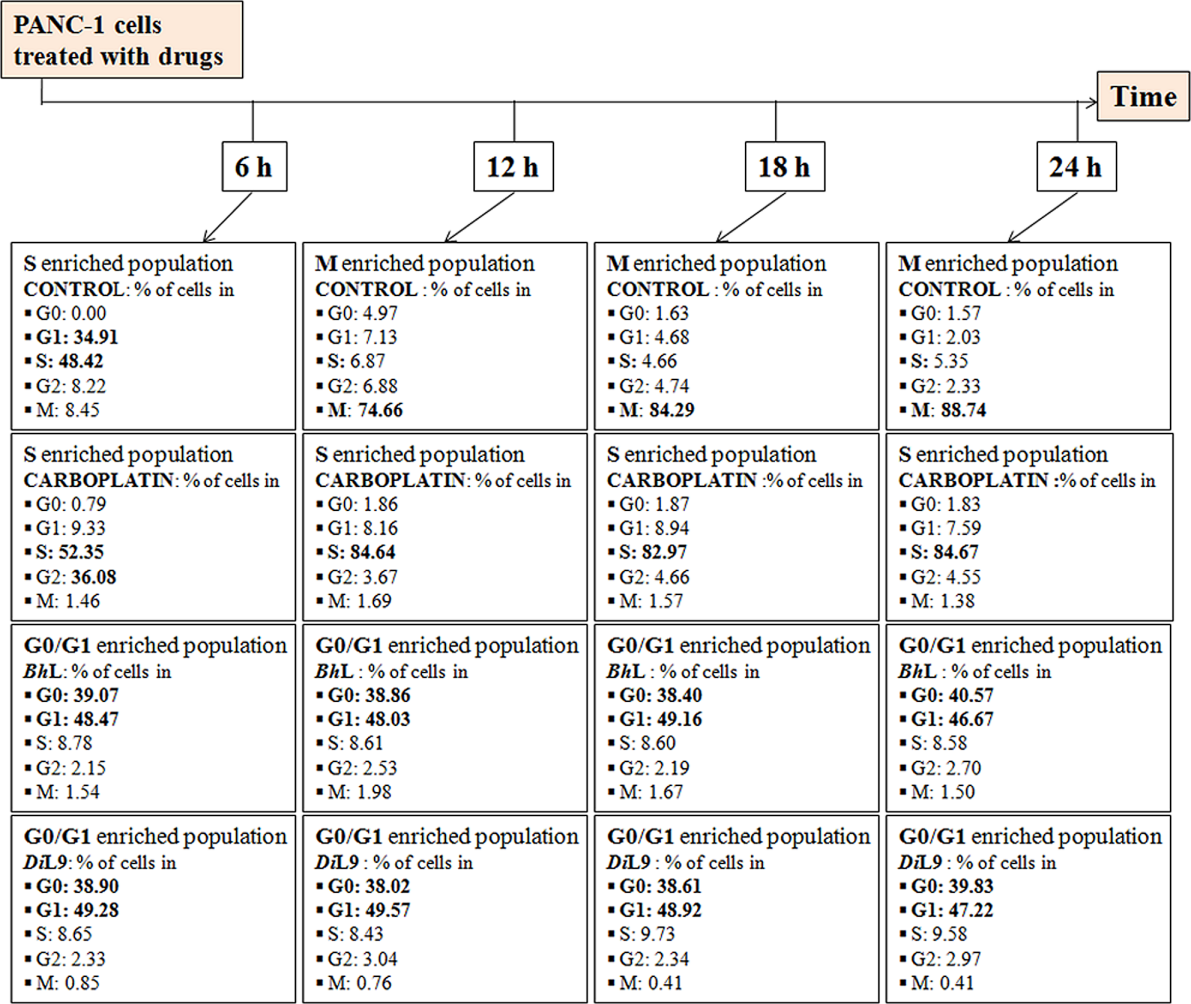
Effect of *Bh*L and *Di*L9 on different phases of cell cycle. The progressive cell cycle changes were observed with DAPI staining after 6, 12, 18 and 24 h on lectin (*Bh*L: 0.247 and *Di*L9: 14.8 μM) treatment and analyzed using HCS software. The darkened numbers indicate the percentage of cells arrested in different phases of cell cycle. The data is mean ± SD of three independent experiments.

To understand the mechanism involved in the cytotoxicity of chito-specific lectins, we first investigated the cell cycle arrest and the lectin-mediated morphological changes in PANC-1 cells. The distribution of cells in different stages of cell cycle was evaluated by exposing the PANC-1 cells to lectins (*Bh*L: 0.247 and *Di*L9: 14.8 μM) for 24 h followed by DAPI staining and HCS analysis. The untreated cells exhibited all the three phases G_0_/G_1_, S and G_2_/M of the cell cycle. Carboplatin treatment showed S phase arrest whereas lectin treated cells showed G_0_/G_1_ arrest with total of 87% populations till 24 h. The effect of both the lectins on PANC-1 cells appeared similar and time-independent, arresting the cells at G_0_/G_1_ phase, with a parallel decrease of the cell population in the S and G_2_/M phase to 8 and 2%, respectively (**Fig 2**).

**Fig 3:**
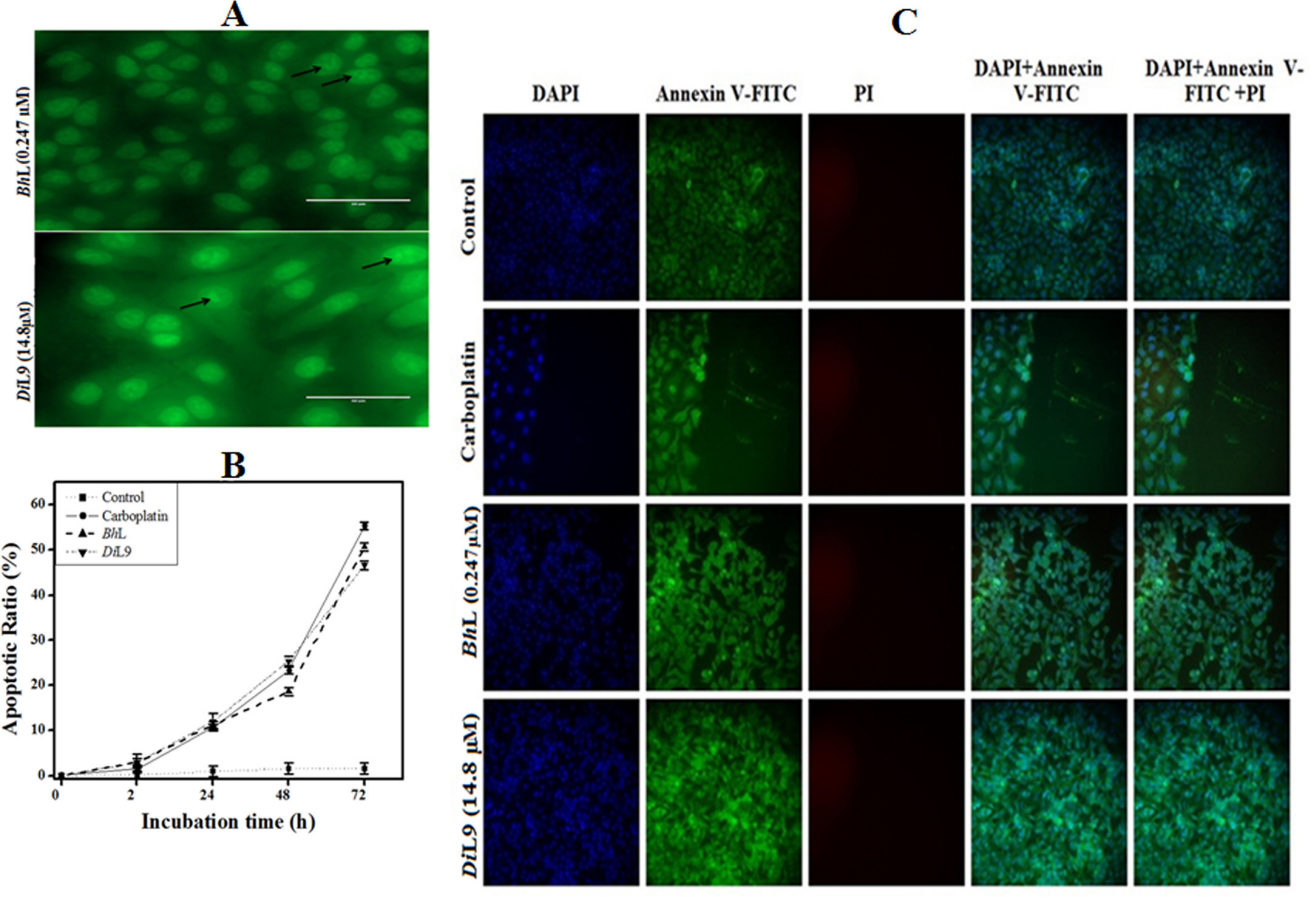
*Bh*L and *Di*L9-induced apoptosis in PANC-1 cells. (A) Acridine orange staining of PANC-1 cells after incubation with lectins (*Bh*L: 0.247 and *Di*L9: 14.8 μM) for 24 h. The arrows indicate apoptotic bodies formed inside the lectin treated cells. Annexin V-FITC/PI staining of lectin stimulated PANC-1 cells incubated for indicated time. (B) The graph represents percentage of cells undergoing apoptosis, mean value ±SD of three independent experiments. (C) The overlay represents the cells that have undergone apoptosis (Annexin V-FITC positive, green) or necrosis (PI positive cells, red) after 72 h of incubation. The analysis was carried out using HCS 2.0 Cell Analysis Software.

Observing changes in the cell morphology is the standard method for recognizing the apoptosis process. Lectin treated cells showed complete disintegration of nuclei and they transformed into small condensed apoptotic bodies observed using AO staining (**Fig 3A**). The apoptotic potential of lectin was quantified using Annexin V-FITC/PI staining, and also differentiates between viable, apoptotic and necrotic cells. There has been a time-dependent increase in Annexin V-FITC positive cells from 12% at 24 h to approximately 20% at 48 h and 50% at 72 h for both the lectins, indicating that these two lectins induce apoptosis in PANC-1 cells. The graph (**Fig 3B**) depicts the percentage of apoptotic cells (Annexin V-FITC positive) after *Bh*L and *Di*L9 exposure over a period of time. Absence of PI-negative cells even after 72 h of incubation following lectin treatment suggests that major cause of chito-specific lectin induced PANC-1 cell death is apoptosis activation and not necrocytosis (**Fig 3C**). Similar results were obtained also in the case of CFPAC-1 and MIA PaCa-2 cell lines on incubating with lectins for 72 h (**S4 and S5 Figs**).

### Activation of intrinsic apoptotic pathway

**Fig 4:**
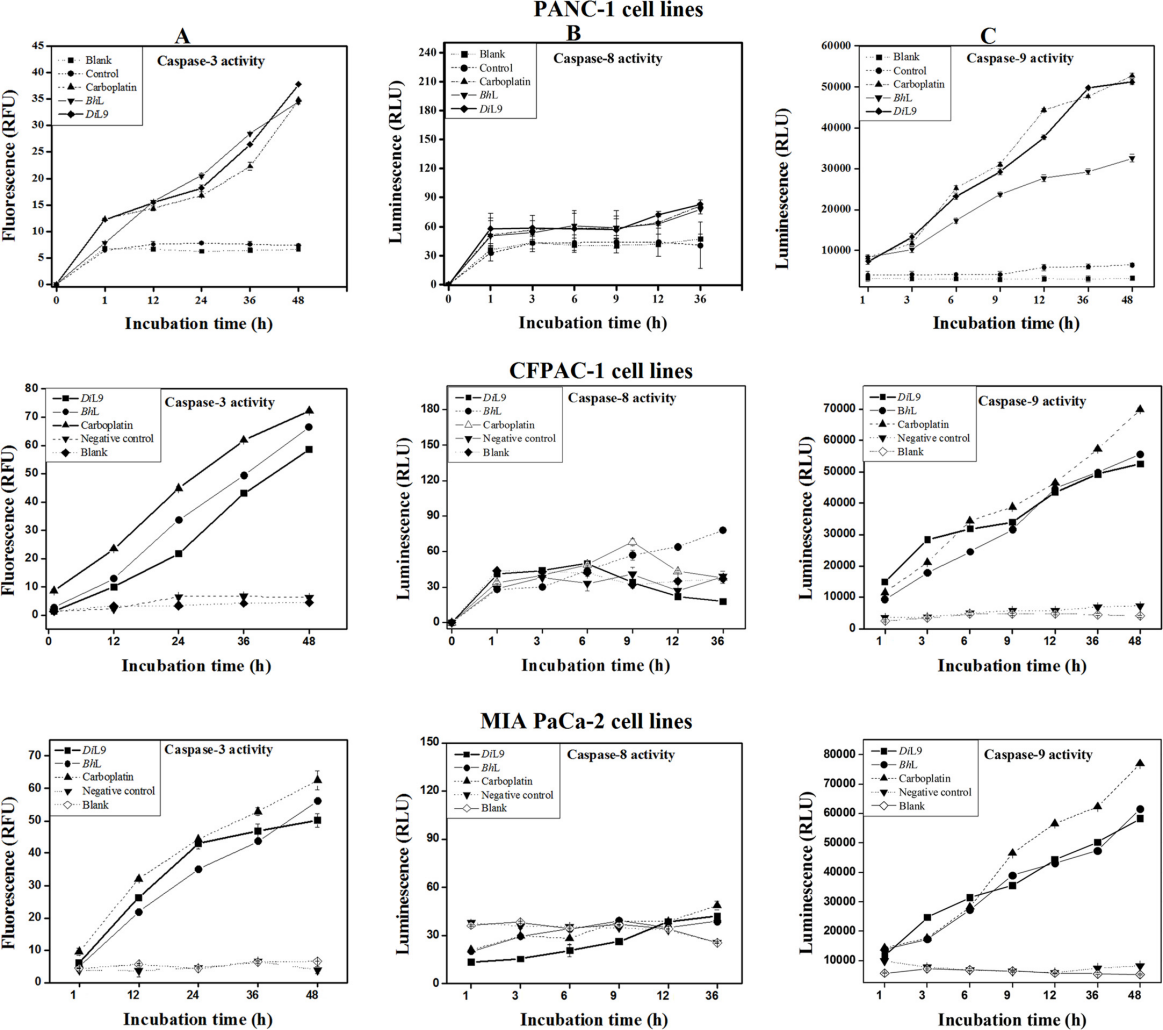
Effect of *Bh*L and *Di*L9 on activation of caspases. The lectin (*Bh*L: 0.247 and *Di*L9: 14.8 μM) treated PANC-1, CFPAC-1 and MIA PaCa-2 cells were incubated for different time periods and activities of caspases were assessed by fluorimetric assay. (A) Caspase-8, (B) caspase-9 and (C) caspase-3 activity was measured with respect to untreated cells. This data is mean ± SD values from three independent experiments.

To analyze the details of lectin-induced apoptosis in PANC-1, CFPAC-1 and MIA PaCa-2 cells, the effect of *Bh*L and *Di*L9 on activation of initiator caspases-8 and-9 involved in apoptotic pathway was evaluated. The time course profiles showed a drastic difference in the caspase-8 and -9 activities. Caspase-8 activity was not detected even after 36 h of lectin treatment (**Fig 4A**) whereas caspase-9 activity increased significantly over a period of time when compared with untreated cells (**Fig 4B**). To confirm this, we analyzed the caspase-8 and -9 activation patterns under the presence of specific caspase inhibitors. Caspase-8/9 inhibitor was added to the cell cultures according to the manufacturer’s instructions to completely inhibit the enzyme activity. In the presence of caspase-9 inhibitor, induction of apoptosis was prevented. On the other hand, pretreatment with caspase-8 inhibitor could not abolish cell death. The results clearly established that only caspase-9 was activated by both the lectins. A time-dependent increase in caspase-3 proteolytic activity was also observed by *Bh*L and *Di*L9 treatment with 5-fold and 3-fold increase, respectively, after 48 h of incubation when compared with untreated cells (**Fig 4C**). Pretreatment of caspase-3 inhibitor, Ac-DEVD-CHO also prevented lectin-induced cell apoptosis. Caspase-9 activity was detected after 3 h followed that by caspase-3 activity at 12 h, implying activation of caspase-9 prior to caspase-3. This activity profile was found similar to carboplatin response [21, 22]. The aforementioned findings indicate that both the lectins, *Bh*L and *Di*L9 induced caspase-dependent apoptosis in a similar manner where caspase-9 is strongly activated. These observations on the effect of lectins on PANC-1 were found to be similar on CFPAC-1 and MIA PaCa-2 cell lines also **(Fig 4).**

### Perturbation of mitochondrial membrane potential (Δψ_m_)

**Fig 5:**
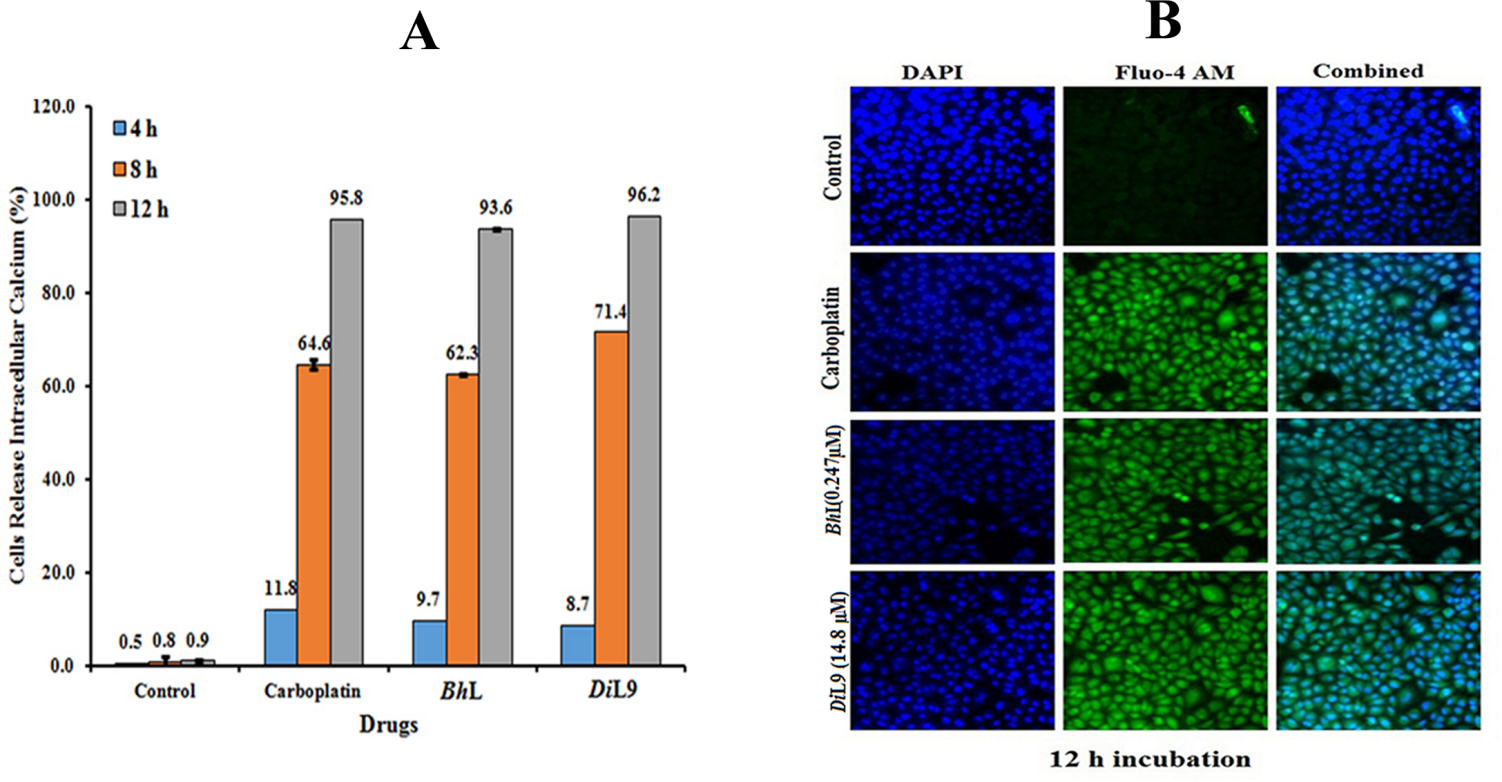
Increase in intracellular [Ca^2+^]i release of lectin stimulated PANC-1 cells. PANC-1 cells were treated with lectins (*Bh*L: 0.247 and *Di*L9: 14.8 μM) for 4, 8 and 12 h, stained with Fluo-4/AM (4μM, green) and DAPI (blue). (A) Depicts the percentage of cells releasing calcium over a period of time. (B) Represents the overlay of confocal microscopy images of fluorescence intensity of cells bound with Fluo-4/AM (green) releasing calcium after 12 h of incubation. The analysis was carried out using LSCM, Magnification 20X (scale, 100 μm).

**Fig 6:**
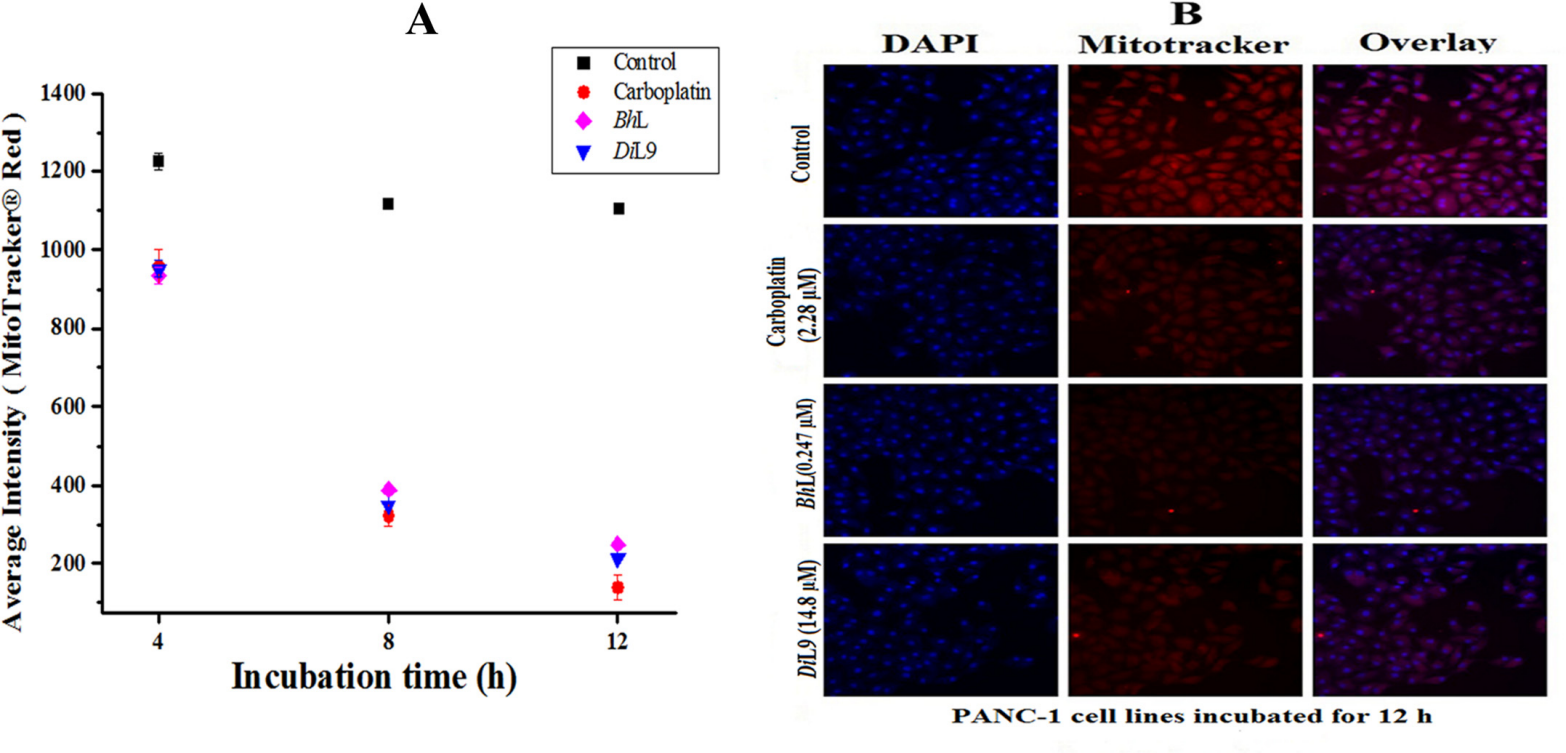
Alteration of mitochondrial transmembrane potential. Lectin (*Bh*L: 0.247 and *Di*L9: 14.8 μM) treated cells were incubated for 4, 8 and 12 h and stained with Mito Tracker Red (0.1 μmol l^-1^) for 15 min at 37°C. (A) Quantification of average intensity of mitochondrial depolarized cells. Data are expressed as mean value ± SD, obtained from three independent experiments.(B) The overlay represents the fluorescence intensity of cells bound with Mitotracker Red, recorded by LSCM, Magnification 20X objective (scale, 100 μm). Loss in red intensity represents the loss in mitochondrial membrane potential.

In the intrinsic pathway of apoptosis, caspase-9 is an initiator enzyme which gets activated by upstream pro-apoptotic signal molecules like cytochrome c and Ca^2+^, released to trigger the cascade of cell death. Increase in the cytoplasmic level of calcium ions is known to secondarily alter mitochondrial homeostasis. The effect of lectins in modulating apoptosis by elevating the [Ca^2+^]_i_ in PANC-1 cells was studied using calcium sensitive dye (Fluo-4 AM). The number of cells releasing calcium increased significantly in a time-dependent manner, measured as 93.6% (*Bh*L), 96.2% (*Di*L9) and 95.8% (Carboplatin), while the control showed only 0.9% even after incubating for 12 h (**Fig 5A and 5B**). Slow accumulation of [Ca^2+^]_i_ in mitochondria leads to its overloading and forms a transition pore, disrupting the Δψ_m_. To evaluate the integrity of mitochondrial membranes, the lectin-treated PANC-1 cells were stained with Mitotracker dye. Treatment with the lectins resulted in the decrease of fluorescent intensity of Mitotracker dye staining indicative of loss of Δψ_m_ in a time-dependent manner from 8 h post-lectin-treatment with further decline occurring at 12 h (**Fig 6A and 6B**). Hence, these results indicate that *Bh*L and *Di*L9 induced cytotoxicity is through caspase-9 dependent apoptosis in which mitochondrial perturbation occurs as upstream events. The observations were similar in the case of other pancreatic cell lines CFPAC-1 and MIA PaCa-2, thus showing mitochondrial dysfunction on treatment with quantity equivalent to GI_50_ values of lectins (**S6 Fig**).

### Lectins showing anti-angiogenesis activity

**Fig 7:**
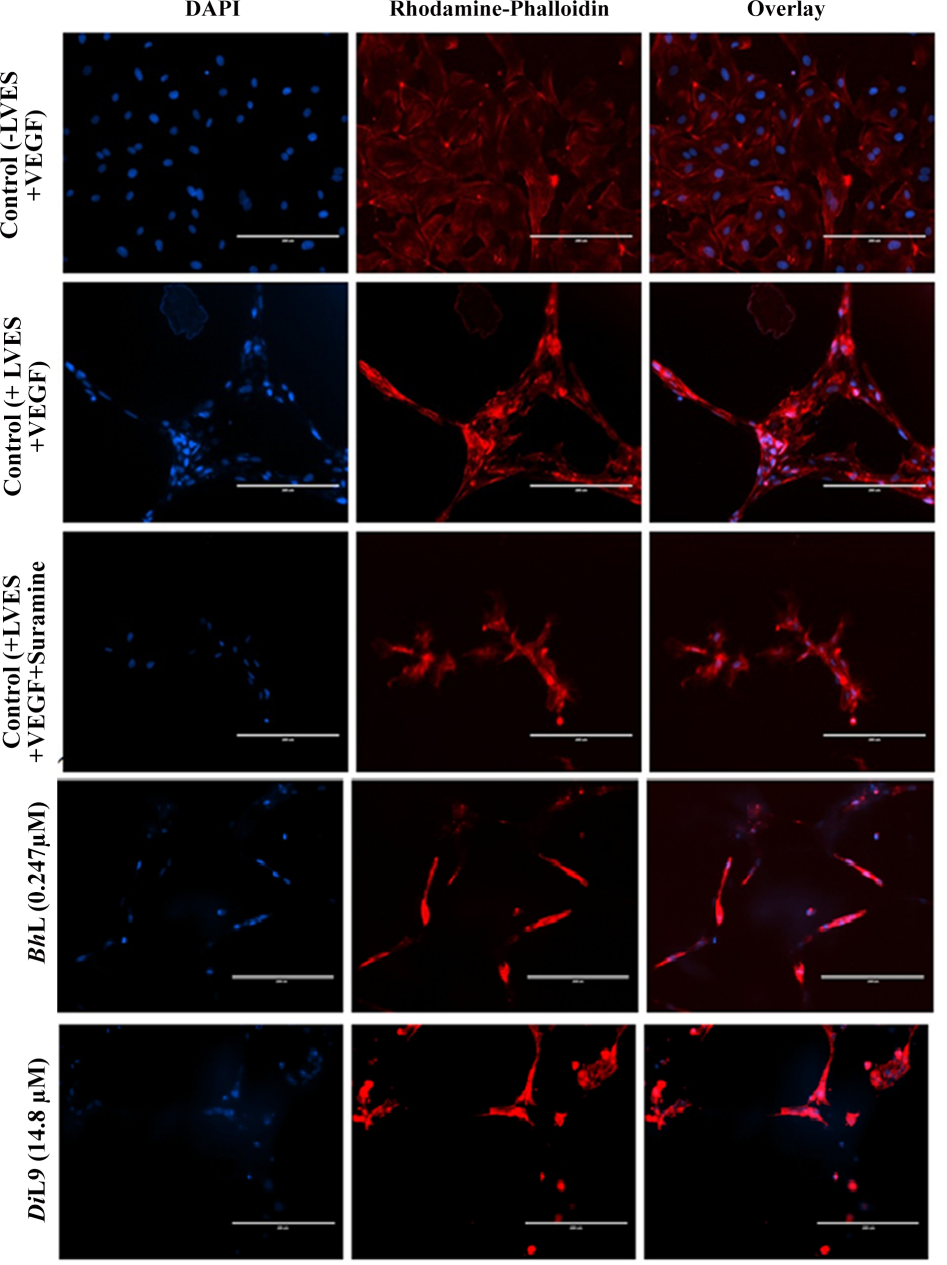
Inhibition of angiogenesis by *Bh*L and *Di*L9. HUVECs were plated on Matrigel (± LVES+VEGF) precoated 96-well plates as control. The cells were treated with lectins. The tubes were stained with rhodamine conjugated phalloidin (red) and nuclei with DAPI (blue). Anti-angiogenic activity is determined by the breakage in the tubule formation. Cell imaging was done on Cellomics’ ArrayScan HCS Reader.

To study the response of adding lectins *Bh*L and *Di*L9 on anti-angiogenesis activity, the HUVECs were treated with lectins. When plated with matrigel, these endothelial cells underwent rapid reorganization, ceased proliferation and formed capillary like tubular structures in the presence of large vessel endothelial supplement (LVES) and VEGF and in its absence the endothelial cells tend to proliferate. Initially, we have shown that both the lectins don’t inhibit the growth of HUVECs as evaluated by MTT assay but surprisingly they could inhibit endothelial tubulogenesis in vitro (**Fig 7**). Both the lectins interrupted in the tube formation in a similar manner, like suramin inhibiting angiogenesis.

## Discussions

Many anticancer agents have been derived from natural sources, including plants, animals, microbes and marine organisms [23]. Due to the property of selectivity and specificity, lectins have gained more attention from researchers in identification of cancer and degree of metastasis [2]. In this study, we have investigated the anticancer activity of two lectins, *Bh*L and *Di*L9, purified from different families of plant sources. Both these lectins showed chito-oligosaccharide specificity, specifically distinct affinity for chitotriose, but widely vary in their structures. As a result they showed similar response against human neoplastic cell lines but differ in terms of dose-response to activity. Even though many lectins have been purified and reported to have wide range of applications, there are only few studies that refer to their anti-cancer potential or reporting on their underlying mechanism of cytotoxicity. Our study showed that the lectins *Bh*L and *Di*L9 displayed cytotoxicity against all human cancer cell lines, with minimum GI_50_ values for pancreatic cancer cells (PANC-1). *Bh*L is found to be more active compared to *Di*L9. Both the lectins showed hemagglutination inhibition activity when incubated with mucin, where mucin-like glycans are commonly over-expressed on 90% of tumors [24]. This clearly shows that the binding of the lectins to the glycoproteins/receptor present on tumor cells appear to be the necessary step for action. Considering example of mucin glycoproteins (MUC1), it has been reported that each pancreatic cancer cell line expresses a unique pattern of MUC1 glycoforms [25]. So binding of these complex lectins to PANC-1, CFPAC-1 and MIA PaCa-2 selectively could provide more accurate analyses of glycans present on these tumor cells and could provide more insight for the treatment of pancreatic cancer effectively. For normal cell lines like HUVECs and L929, *Bh*L and *Di*L9 were not cytotoxic even at higher concentrations (> 200 μg ml^-1^), implying its exclusive specificity for cancerous cells.

**Fig 8:**
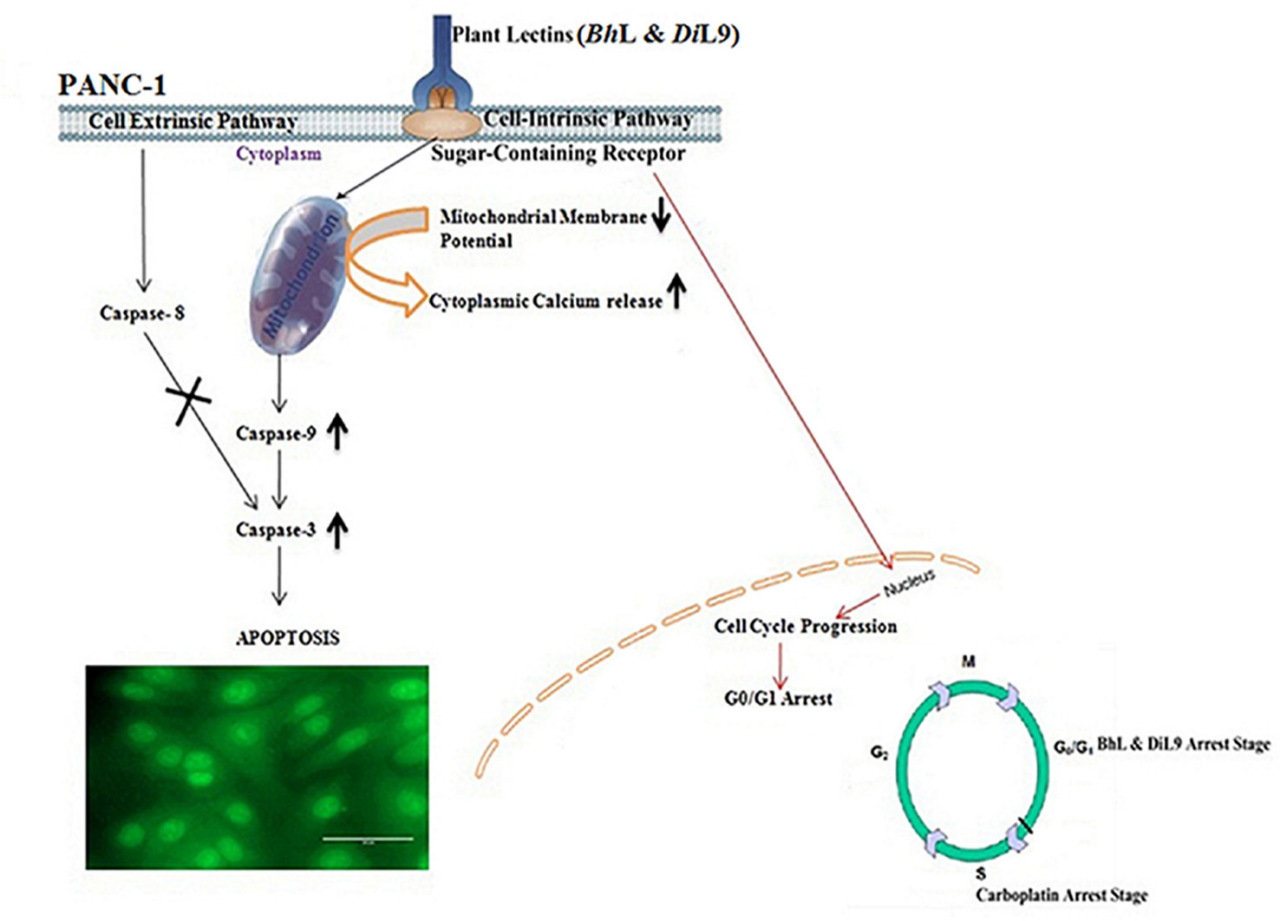
Proposed model for mechanism of apoptosis induced by *Bh*L and *Di*L9 in PANC-1 cells. Lectins bind to the glycoprotein receptors of PANC-1 cells and triggers mitochondrial membrane depolarization cascade. The apoptotic signal was amplified by activation of caspase-9 and -3 leading to final cell death.

To develop lectin-based anti-cancer treatment it is important to understand the underlying mechanism involved during human tumor cell apoptosis induced by lectins [4]. The proposed underlying mechanism of these lectin-induced (*Bh*L and *Di*L9) pancreatic cell death is depicted in **Fig 8**. In order to understand the mechanism of lectin’s antiproliferative effect on tumor cells, morphological changes, activation of caspases, release of calcium ions and mitochondrial membrane depolarization studies were conducted. As mentioned before, PCD, a process that eliminates improperly developed cells, has become the plausible strategy for cancer therapy. The agents targeting apoptotic pathway, specifically in tumor cells, have potential to be useful for antitumor therapy [26]. Shown in this investigation is that *Bh*L and *Di*L9 induce apoptosis strongly against PANC-1 cells than in normal cells, by which they would fit into the category of such anticancer agents.

The morphological analysis by Annexin V-FITC/PI staining demonstrated that the lectin treated cells predominantly undergo apoptosis and not necrosis. When a cell undergoes apoptosis, phosphatidylserine (PS) which usually present on the inner side of the membrane gets exposed on the surface, acting as a key marker for apoptotic bodies to get recognized by phagocytic cells [27]. It is also widely known that the antitumor or DNA damaging agents induce cell death by arresting the cell cycle. In normal cells, the cell cycle checkpoints ensure damage repair, whereas in malignant cells the apoptosis eliminates them. Our study demonstrated that PANC-1 cells treated with *Bh*L and *Di*L9 showed cell death by arresting the cells at G_0_/G_1_ phase of the cell cycle.

Among the caspases that belong to cysteine proteases family, caspase-3 plays an important role in causing apoptosis after its activation by stimulation from cell death receptors (extrinsic) or mitochondria (intrinsic) [28, 29]. For caspases to get activated, the upstream proapoptotic signal molecules like cytochrome c and Ca^2+^ should be released to trigger the cascade of cell death. Thus, mitochondrial membrane potential and release of calcium ions was also evaluated. After 12 h incubation of lectin treated PANC-1 cells, a significant increase in the number of cells releasing Ca^2+^ was observed with severely disrupting mitochondrial membrane potential resulting in the release of cytochrome c which formed apoptosome complex and activated caspase-9. This further led to the activation of caspase-3 leading to final cell death. Hence, these results indicate that cytotoxicity of *Bh*L and *Di*L9 is induced through caspase-9 dependent apoptosis in which mitochondrial perturbation occurs as upstream events. Unlike previously reported lectins such as ConA and WGA [30–32], *Bh*L, among the two lectins studied here, is more potent because of its ability to induce cell death at very low concentrations and activating early intrinsic apoptotic cascade.

Another important factor for the spread of tumor is neovascularization. Tumor-induced angiogenesis is an integral part of maintaining tumor growth and progression by providing the necessary blood supply and also allowing metastatic cells to go into circulation [33]. Angiogenesis inhibitors can block any of the steps in the angiogenic cascade, including endothelial cells proliferation and attachment to the extracellular matrix proteins, migration and invasion through the matrix to form a thin tube meshwork [34]. Thus, angiogenesis also becomes an interesting target for developing anti tumor therapies. Currently, in many laboratories natural and synthetic inhibitors of angiogenesis are studied extensively [35, 36]. For instance, many inhibitors are peptides derived from snake venom like disintegrins [37] and lebectins [38]. So, to evaluate the anti-angiogenic activity of *Bh*L and *Di*L9, HUVEC tube formation assay was used. It is a well established and simple in *vitro* angiogenesis assay based on the ability of endothelial cells to form three-dimensional capillary-like tubular structures that form on matrigel composed of growth factor-reduced basement membrane extracts. Here, both the lectins efficiently inhibited the tubulogenesis process without affecting the viability of confluent HUVECs, also confirmed by MTT assay. So far as we know, there are no reports of chito-specific lectin possessing anti-angiogenic activity at such a low lectin concentration. *Viscum album* extracts inhibits angiogenesis by inducing apoptosis in endothelial cells [39] and ConA targets anti-angiogenesis pathway at 25 μg ml^-1^ [40, 41] whereas *Bh*L at 8 μg ml^-1^ (247 μM) shows much more effective response. This effect of lectins might be due to blocking of the co-receptor binding site of growth factors on endothelial cells, inhibiting its adherence to the matrigel, and thus disrupting the tube formation by endothelial cells for angiogenesis. Therapies for brain tumors are the most risky, where these lectins as anti-angiogenic agents can be the hope for such treatments. According to our present results, we suggest that *Bh*L and *Di*L9 lectins could be used with high efficiency for the inhibition of the brain angiogenic process without any side effects and warrants further investigations like in *vivo* studies.

Previously, using mistletoe lectins many researchers have conducted *in vivo* experiments on different animal models and had reported reduction in tumor size and growth when injected intratumorally [42]. Mostly, these i*n vivo* investigations on the ability of lectins to inhibit cancer cell proliferation in animal models have given inconsistent results due to factors such as heterogeneity of tested animal models, difference in route of administration and sample size. During further investigations, when carrying out clinical trials, many limitations have been reported such as registration of small number of patients and lack of proper control group. Taking into consideration the applications of our reported lectins being non-toxic to normal cells, oral or intratumoral administration might be a promising alternative therapy for pancreatic cancer patients. Further qualitative *in vivo* and clinical trials evaluating the effect of lectins on pancreatic cancer patients must be carried out addressing safety parameters, standard dosage, and appropriate endpoint measures. These studies should take into account the sample size of patients, dosage limit, time of administration and methodological design to prevent failures encountered during the trial of previously tried lectins.

## Conclusions

In summary, we report for the first time that two chito-oligosaccharide specific lectins, *Bh*L and *Di*L9 belonging to different family origin, possess remarkable antiproliferative activity. Among these two lectins, *Bh*L gave much more effective cytotoxicity response than *Di*L9. Both lectins induced apoptosis in human pancreatic cancer cells in a similar manner. The underlying apoptotic mechanism was through the intrinsic pathway causing depolarization of mitochondrial membrane potential and increase in intracellular calcium leading to activation of executioner caspases (caspases-9 and -3). Interestingly, both the lectins also inhibited endothelial tubulogenesis. Our results would open exploration of plant dietary lectins combining them with slow-release and point-insert biomaterials as potential novel candidates for pharmaceutical exploitation. Hence, the successful therapy will be based on the selective elimination of the abnormal cells without disturbing the function of the normal cells.

## Supporting Information

S1 Fig Purity check of lectins on 12% SDS-PAGE. (A) *Bh*L. Lane 1: chitin affinity column fraction; 2- loaded on Sephacryl S-200 column; 3-Protein molecular marker;4-6- Pure *Bh*L. (B) Lane 2: crude extract (homogenate); 3–60% saturation ammonium sulfate-precipitated fraction; 4- dialysed ammonium sulfate fraction; 5-unbound fraction from Q-sepharose column; 6-Biorad broad range protein molecular weight marker; 7- Pure *Di*L9 after Sephacryl S-200 column.
(TIF)

S2 Fig Anti-proliferative activity of *Bh*L and *Di*L9 on CFPAC-1 and MIA PaCa-2 cells: The cancer cell lines were treated with serial dilutions of lectins and incubated for 48 h. The growth inhibition (%) was measured by MTT assay by considering untreated cells as 100%. (A) Effect of *Bh*L and (B) *Di*L9 treatment on above mentioned cell lines. The values presented in the graph are the mean ± SD of two independent experiments done in triplicates.
(TIF)

S3 Fig Effect of serum incubated lectin on cancer cells: Both the lectins (*Bh*L and *Di*L9) were incubated with 100% serum for 24 h. MTT assay was carried out to determine the effect of serum incubated lectin on pancreatic cancer cell lines (PANC-1, CFPAC-1 and MIA PaCa-2). The values depicted in the graph are the mean ± SD of three independent experiments done.
(TIF)

S4 Fig Apoptotic effects of *Bh*L and *Di*L9 on CFPAC-1 cells. The human pancreatic CFPAC-1 cells were incubated with or without lectins (*Bh*L and *Di*L9, GI_50_ conc) for 72 h. The cells were stained with DAPI, Annexin V-FITC and PI. The overlay represents the cells that have undergone apoptosis (Annexin V-FITC positive, green) or necrosis (PI positive cells, red).The analysis was carried out using HCS 2.0 Cell Analysis Software.
(TIF)

S5 Fig Apoptotic effects of *Bh*L and *Di*L9 on MIA PaCa-2 cells. The human pancreatic MIA PaCa-2 cells were incubated with or without lectins (*Bh*L and *Di*L9, GI_50_ conc) for 72 h. The cells were stained with DAPI, Annexin V-FITC and PI. The overlay represents the cells that have undergone apoptosis (Annexin V-FITC positive, green) or necrosis (PI positive cells, red).The analysis was carried out using HCS 2.0 Cell Analysis Software.
(TIF)

S6 Fig Effect of lectins on MMP of CFPAC-1 and MIA PaCa-2 cells: Lectin treated cells were incubated for 12 h. The mitochondria were red with Mito Tracker Red (0.1 μmol l^-1^) and nuclei were stained with DAPI (1 μmol l^-1^) for 15 min at 37°C. Decrease in red intensity indicates loss in MMP. The images have been recorded by LSCM, Magnification 20X objective (scale, 100 μm).
(TIF)

## Abbreviations

BhL: *Benincasa hispida* lectin
DiL9: *Datura innoxia* lectin
DAPI: 4′,6-Diamidino-2-phenylindole
FITC: Fluorescein isothiocyanate
HCS: High content screening
LSCM: laser-scanning confocal microscope
MTT: 3,4, 5-dimethylthiazol-2-yl-2, 5- diphenyltetrazolium bromide
PI: Propium iodide
