# Supplementary figures and images for: Two Chitotriose-Specific Lectins Show Anti-Angiogenesis, Induces Caspase-9-Mediated Apoptosis and Early Arrest of Pancreatic Tumor Cell Cycle
Source: PLoS One. 2016 Jan 21;11(1):e0146110. doi: 10.1371/journal.pone.0146110 (PMC4721955; doi:10.1371/journal.pone.0146110)

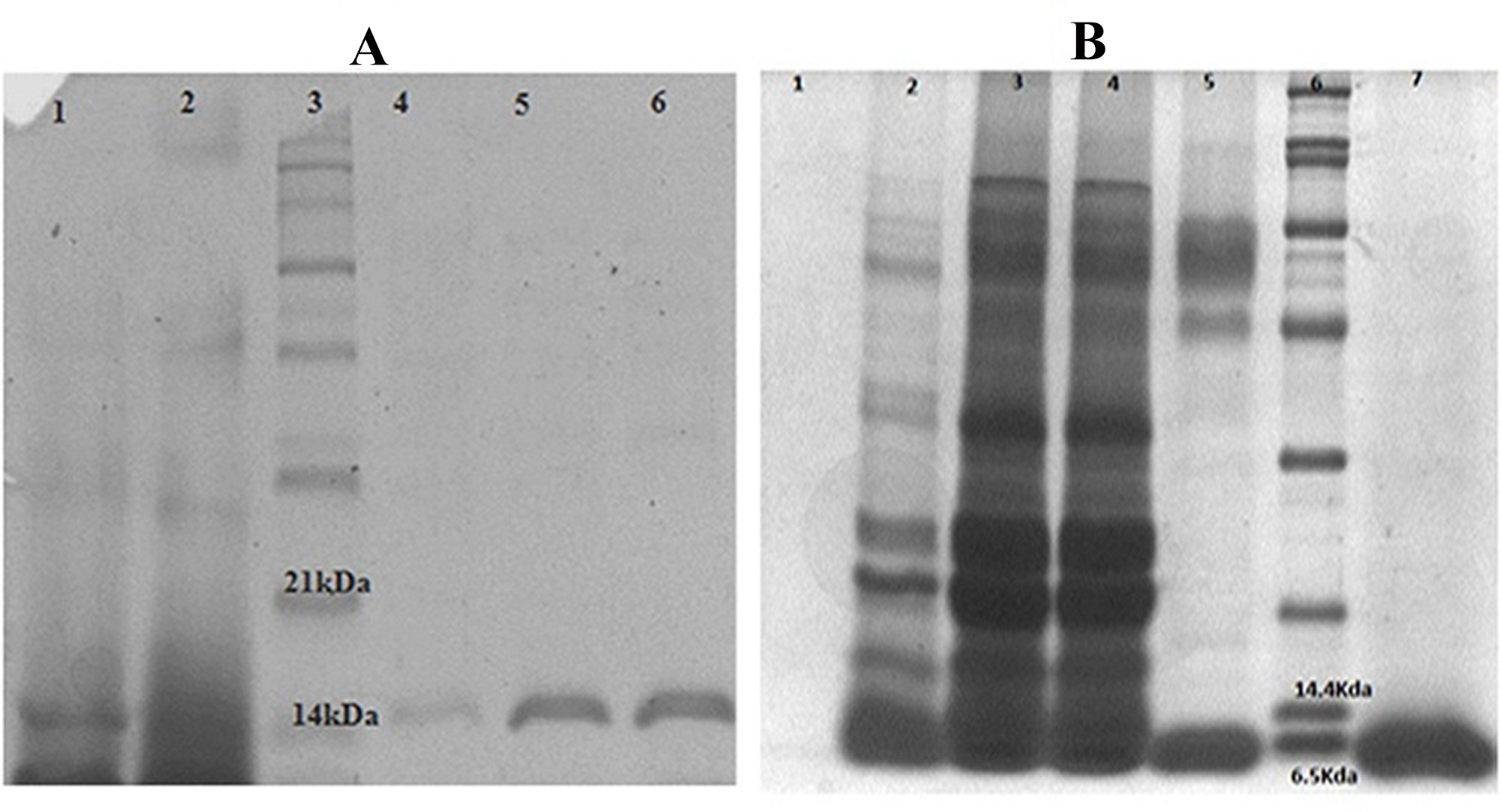

Supplement: S1 Fig — (A) BhL. Lane 1: chitin affinity column fraction; 2- loaded on Sephacryl S-200 column; 3-Protein molecular marker;4-6- Pure BhL. (B) Lane 2: crude extract (homogenate); 3–60% saturation ammonium sulfate-precipitated fraction; 4- dialysed ammonium sulfate fraction; 5-unbound fraction from Q-sepharose column; 6-Biorad broad range protein molecular weight marker; 7- Pure DiL9 after Sephacryl S-200 column. (TIF) [file pone.0146110.s001.tif]

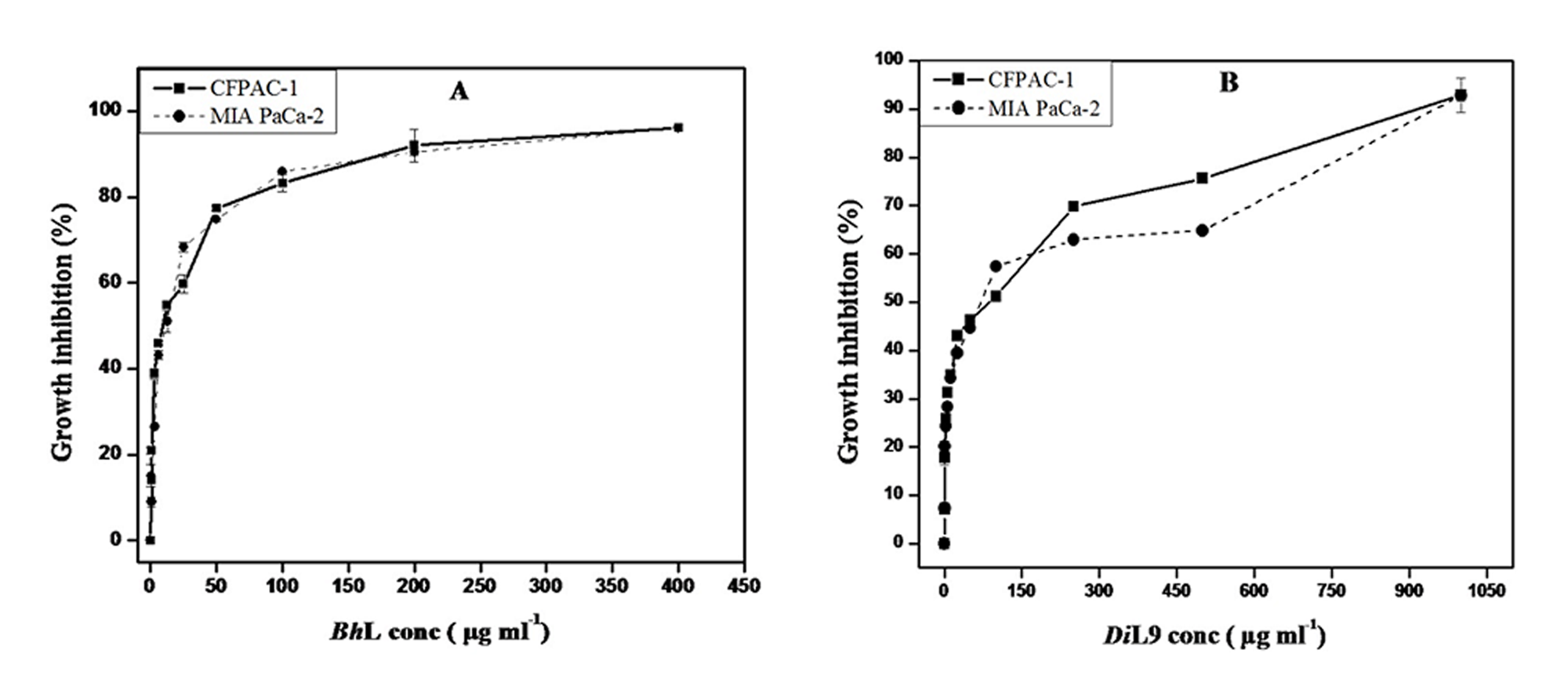

Supplement: S2 Fig — The growth inhibition (%) was measured by MTT assay by considering untreated cells as 100%. (A) Effect of BhL and (B) DiL9 treatment on above mentioned cell lines. The values presented in the graph are the mean ± SD of two independent experiments done in triplicates. (TIF) [file pone.0146110.s002.tif]

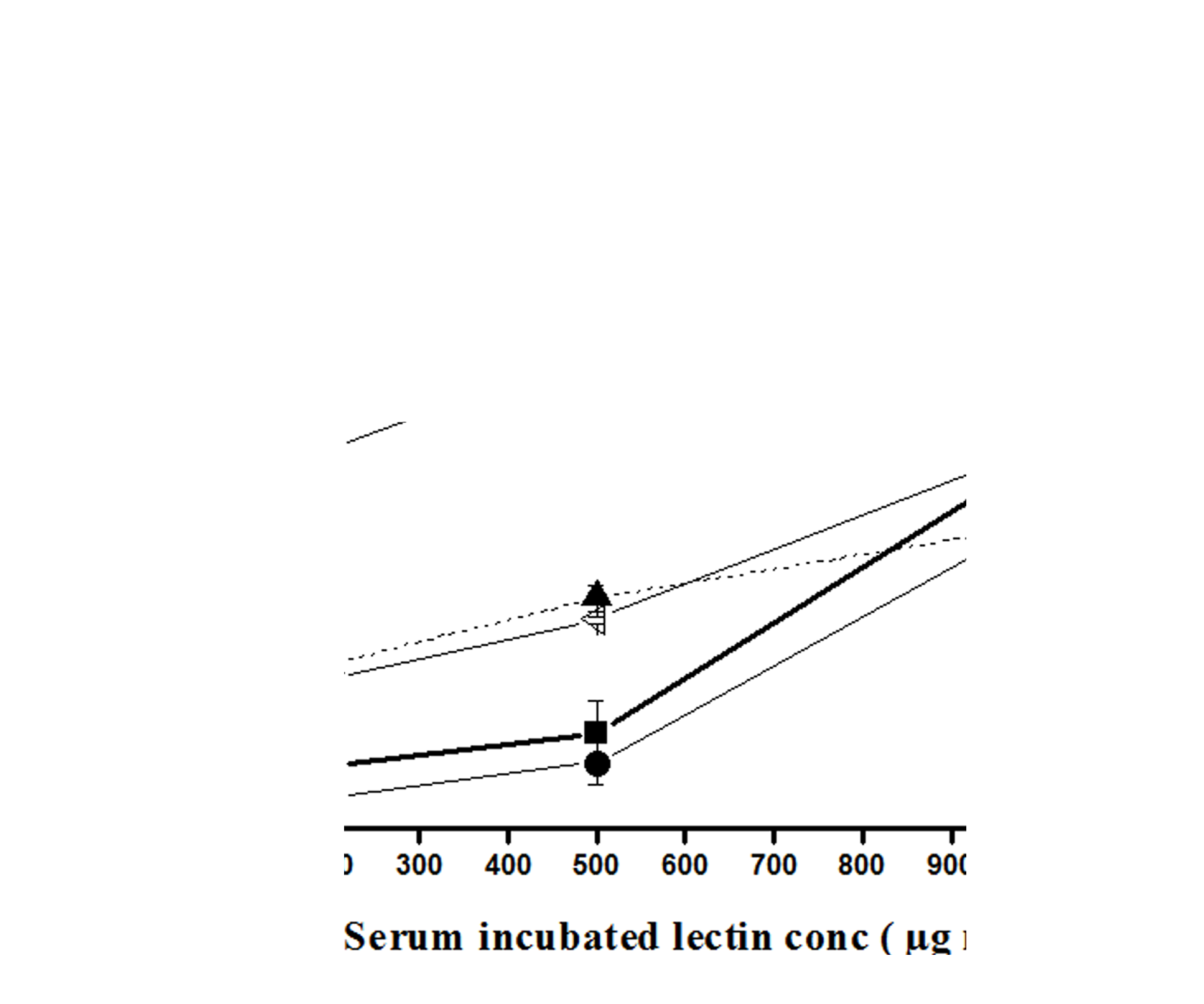

Supplement: S3 Fig — MTT assay was carried out to determine the effect of serum incubated lectin on pancreatic cancer cell lines (PANC-1, CFPAC-1 and MIA PaCa-2). The values depicted in the graph are the mean ± SD of three independent experiments done. (TIF) [file pone.0146110.s003.tif]

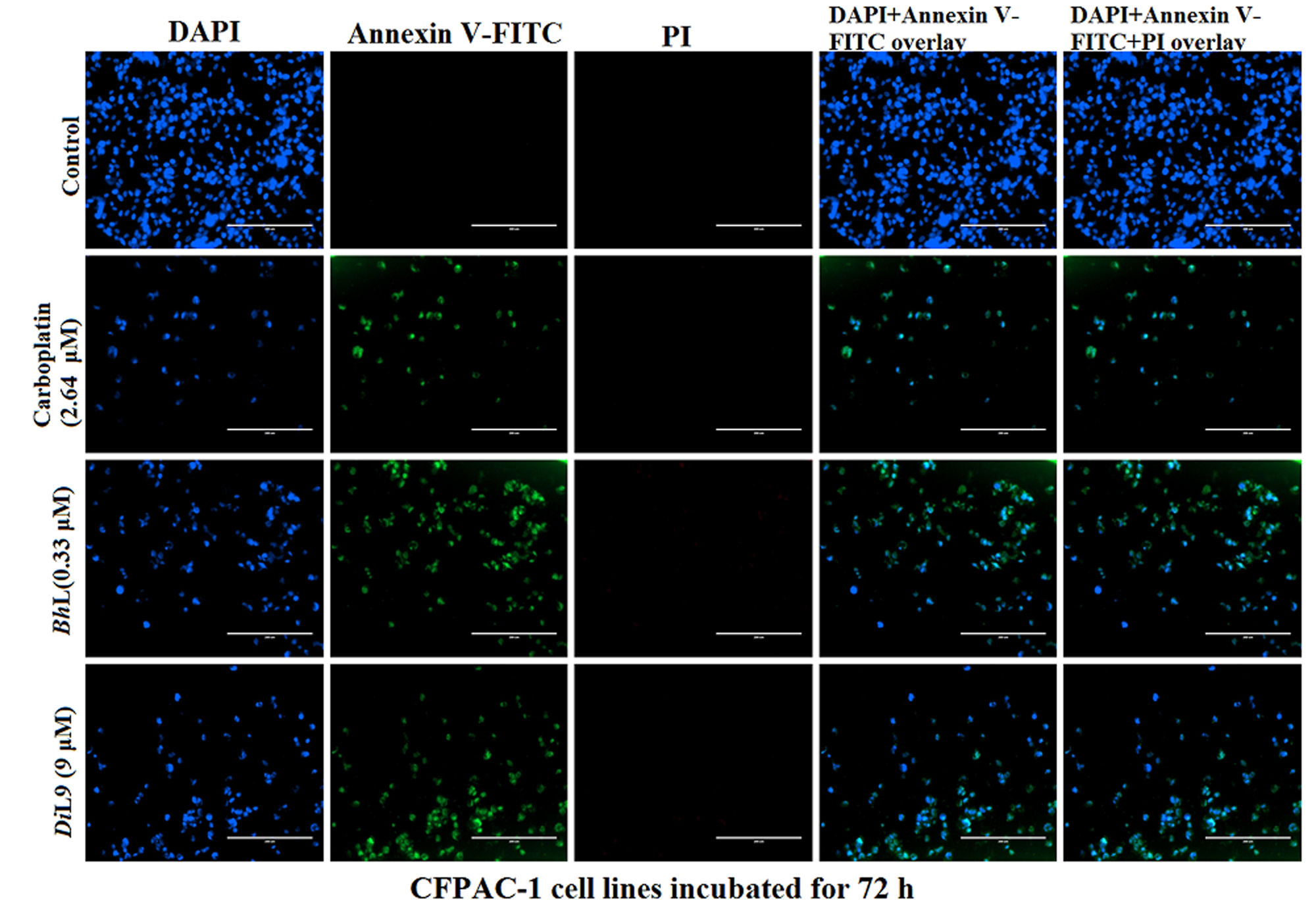

Supplement: S4 Fig — The human pancreatic CFPAC-1 cells were incubated with or without lectins (BhL and DiL9, GI50 conc) for 72 h. The cells were stained with DAPI, Annexin V-FITC and PI. The overlay represents the cells that have undergone apoptosis (Annexin V-FITC positive, green) or necrosis (PI positive cells, red).The analysis was carried out using HCS 2.0 Cell Analysis Software. (TIF) [file pone.0146110.s004.tif]

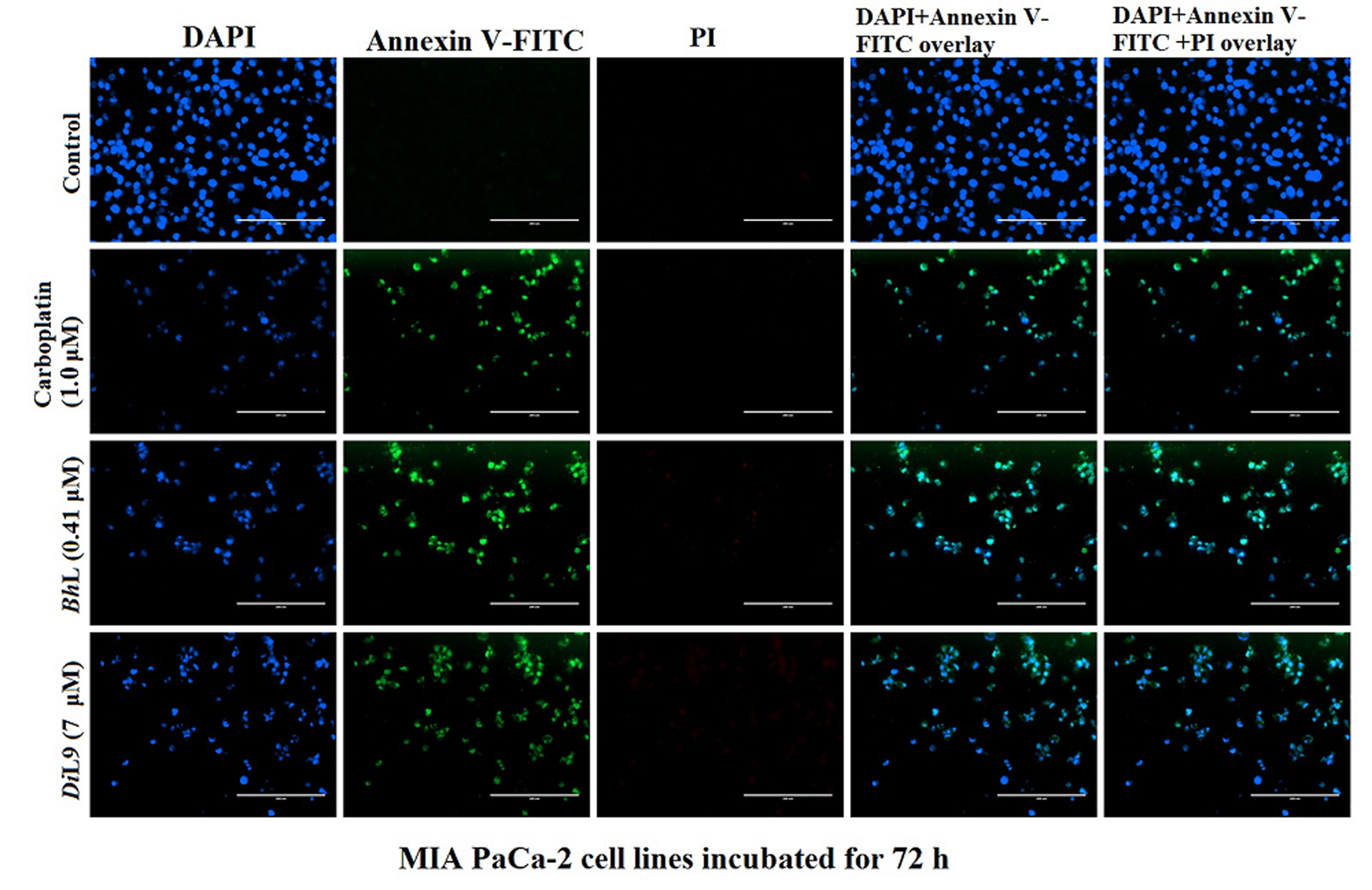

Supplement: S5 Fig — The human pancreatic MIA PaCa-2 cells were incubated with or without lectins (BhL and DiL9, GI50 conc) for 72 h. The cells were stained with DAPI, Annexin V-FITC and PI. The overlay represents the cells that have undergone apoptosis (Annexin V-FITC positive, green) or necrosis (PI positive cells, red).The analysis was carried out using HCS 2.0 Cell Analysis Software. (TIF) [file pone.0146110.s005.tif]

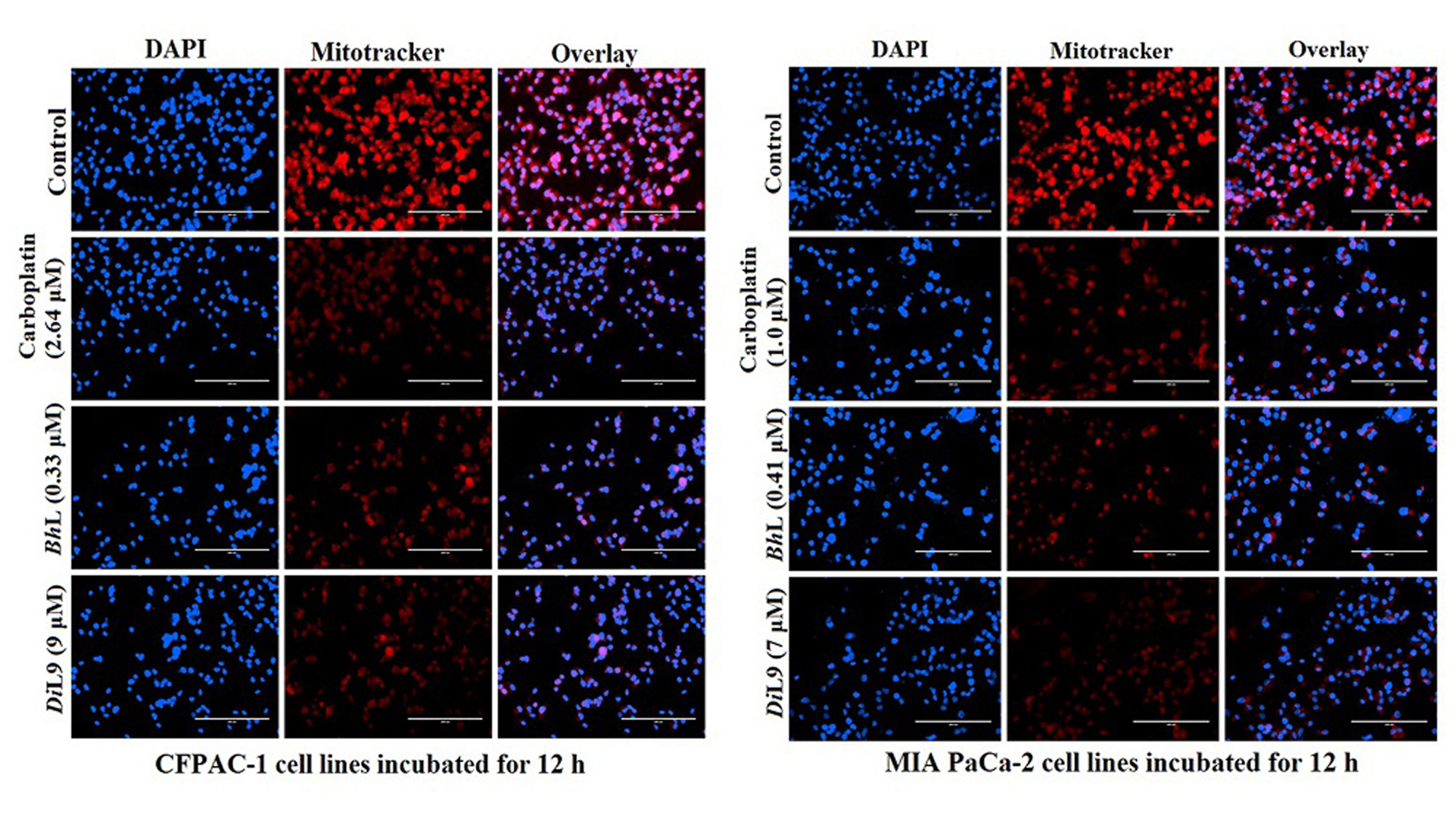

Supplement: S6 Fig — The mitochondria were red with Mito Tracker Red (0.1 μmol l-1) and nuclei were stained with DAPI (1 μmol l-1) for 15 min at 37°C. Decrease in red intensity indicates loss in MMP. The images have been recorded by LSCM, Magnification 20X objective (scale, 100 μm). (TIF) [file pone.0146110.s006.tif]
